# Bi-directional signalling between the intestinal epithelium and type-3 innate lymphoid cells (ILC3) regulates secretory dynamics and IL-22

**DOI:** 10.1016/j.mucimm.2023.11.002

**Published:** 2023-11-10

**Authors:** Emily Read, Ainize Peña-Cearra, Diana Coman, Geraldine M. Jowett, Matthew W H Chung, Isabelle Coales, Sofia Syntaka, Rachel E. Finlay, Roser Tachó-Piñot, Sjoerd van Der Post, Umar Naizi, Luke B. Roberts, Matthew R. Hepworth, Michael A. Curtis, Joana F. Neves

**Affiliations:** 1 Centre for Host Microbiome Interactions, King’s College London, London UK; 2 Wellcome Trust Advanced Therapies and Regenerative Medicine PhD Programme, London, UK; 3 Department of Immunology, Microbiology and Parasitology, Faculty of Medicine and Nursing, University of the Basque Country (UPV/EHU), Spain; 4 Wellcome Trust/Cancer Research UK Gurdon Institute, University of Cambridge, Tennis Court Road, Cambridge CB2 1QN, UK; 5 Centre for Gene Therapy & Regenerative Medicine, King´s College London, UK; 6 Peter Gorer Department of Immunobiology, School of Immunology and Microbial Sciences, King’s College London, London, UK; 7 Lydia Becker Institute of Immunology and Inflammation, Division of Infection, Immunity and Respiratory Medicine, Faculty of Biology, Medicine and Health, the University of Manchester, Manchester Academic Health Science Centre. Manchester, UK; 8 Department of Medical Biochemistry and Cell biology, University of Gothenburg, Gothenburg, Sweden; 9 Guy’s and St Thomas’ National Health Service Foundation Trust and King’s College London National Institute for Health Research and Social Care, Biomedical Research Centre Translational Bioinformatics Platform, Guy’s Hospital, London UK

## Abstract

Type-3 innate lymphoid cells (ILC3) respond to localised environmental cues to regulate homeostasis and orchestrate immunity in the intestine. The intestinal epithelium is an important upstream regulator and downstream target of ILC3 signalling, however the complexity of mucosal tissues can hinder efforts to define specific interactions between these two compartments. Here, we employ a reductionist co-culture system of murine epithelial small intestinal organoids (SIO) with ILC3 to uncover bi-directional signalling mechanisms that underlie intestinal homeostasis. We report that ILC3 induce global transcriptional changes to intestinal epithelial cells, driving the enrichment of secretory goblet cell signatures. We find that SIO enriched for goblet cells promote NKp46^+^ ILC3 and IL-22 expression, which can feedback to induce IL-22-mediated epithelial transcriptional signatures. However, we show epithelial regulation of ILC3 in this system is contact-dependent and demonstrate a role for epithelial Delta-Like-Canonical-Notch-Ligand (Dll) in driving IL-22 production by ILC3, via subset-specific Notch1-mediated activation of T-bet^+^ ILC3. Finally, by interfering with Notch ligand-receptor dynamics, ILC3 appear to upregulate epithelial *Atoh1* to skew secretory lineage determination in SIO-ILC3 co-cultures. This research outlines two complimentary bi-directional signalling modules between the intestinal epithelium and ILC3, which may be relevant in intestinal homeostasis and disease.

## Introduction

Type-3 innate lymphoid cells (ILC3) are crucial players in the intestinal immune landscape^1^. Although unified by expression of interleukin-7 receptor subunit-α (IL-7Rα) and the transcription factor RAR-related orphan receptor (ROR)γt in the absence of other myeloid and lymphoid markers, ILC3 in the postnatal intestine are heterogenous and subsets include Natural Cytotoxicity Receptor (NCR)^+^ cells, CCR6^+^ CD4^+/-^ Lymphoid tissue inducer (LTi)- like cells and “double negative” (DN) populations which lack NCR expression and LTi-like markers^2^. Among these populations, NCR^+^ ILC3 and DN cells can express the type 1 transcription factor T-bet. These subsets are plastic, with redundant and non-redundant roles in the intestinal mucosa, including supporting barrier protection^3^, host defence^4^ and adaptive immune cell regulation^5^.

ILC3 are highly sensitive to their surrounding microenvironment^1^. A growing body of literature has identified numerous signalling modules between ILC3 and the intestinal epithelium that are important in a diverse range of homeostatic and inflammatory processes^6^. Much of this research has focused on the impact of ILC3 signals on the epithelium, with IL-22 taking centre stage for pleiotropic, context dependent and subset specific regulation of epithelial cells^7–9^. However, ILC3 can produce other cytokines and signalling factors which may synergise with IL-22 to regulate the epithelium^10,11^. Furthermore, epithelial-ILC3 signalling modules are likely bi-directional, with the intestinal epithelium primely located and highly adapted to sense not only signs of infection and damage, but also homeostatic variations in dietary and metabolic signals^12^. Of particular interest is how epithelial-ILC3 interactions change in inflammatory diseases, such as Inflammatory Bowel Disease (IBD). Hallmark changes to epithelial and ILC3 compartments in IBD include a significant reduction in the proportion and function of secretory goblet cell populations^13–15^ and NCR^+^ ILC3 subsets^16–18^.

To tease apart ILC3-epithelial intestinal interactions, we employed an *in vitro* co-culture system between murine epithelial small intestinal organoids (SIO) and small intestinal lamina propria (SILP) derived ILC3, which allows thorough interrogation of the communication between these compartments in a reductionist setting. This approach has been successfully used to describe the impact of ILC3 derived IL-22 on intestinal epithelial regeneration^19^ and to validate the upregulation of IL-22 target genes in the intestinal epithelium following co-culture with ILC3 activated by neural peptides^20^. We aimed to identify new signalling modules between ILC3 and the intestinal epithelium, and to characterize the impact of those pathways on epithelial and ILC compartments. We identify broad transcriptional changes to intestinal epithelial cells (IEC) by ILC3, notably the enrichment of secretory cell signatures. We then reveal the capacity of goblet cell enriched SIO to robustly maintain NKp46^+^ ILC3 in co-culture and induce expression of IL-22 in a contact dependent fashion. Finally, we outline a role for the Notch pathway in epithelial regulation of IL-22 production in T-bet^+^ ILC3 and the enrichment of secretory epithelial signatures by ILC3. This work dissects a critical component of the complicated network of signalling between the intestinal epithelium and ILC3, which is relevant in homeostatic intestinal function and may be disrupted in disease.

## Results and Discussion

### ILC3 promote epithelial secretory cell signatures in SIO co-cultures

**Figure 1:**
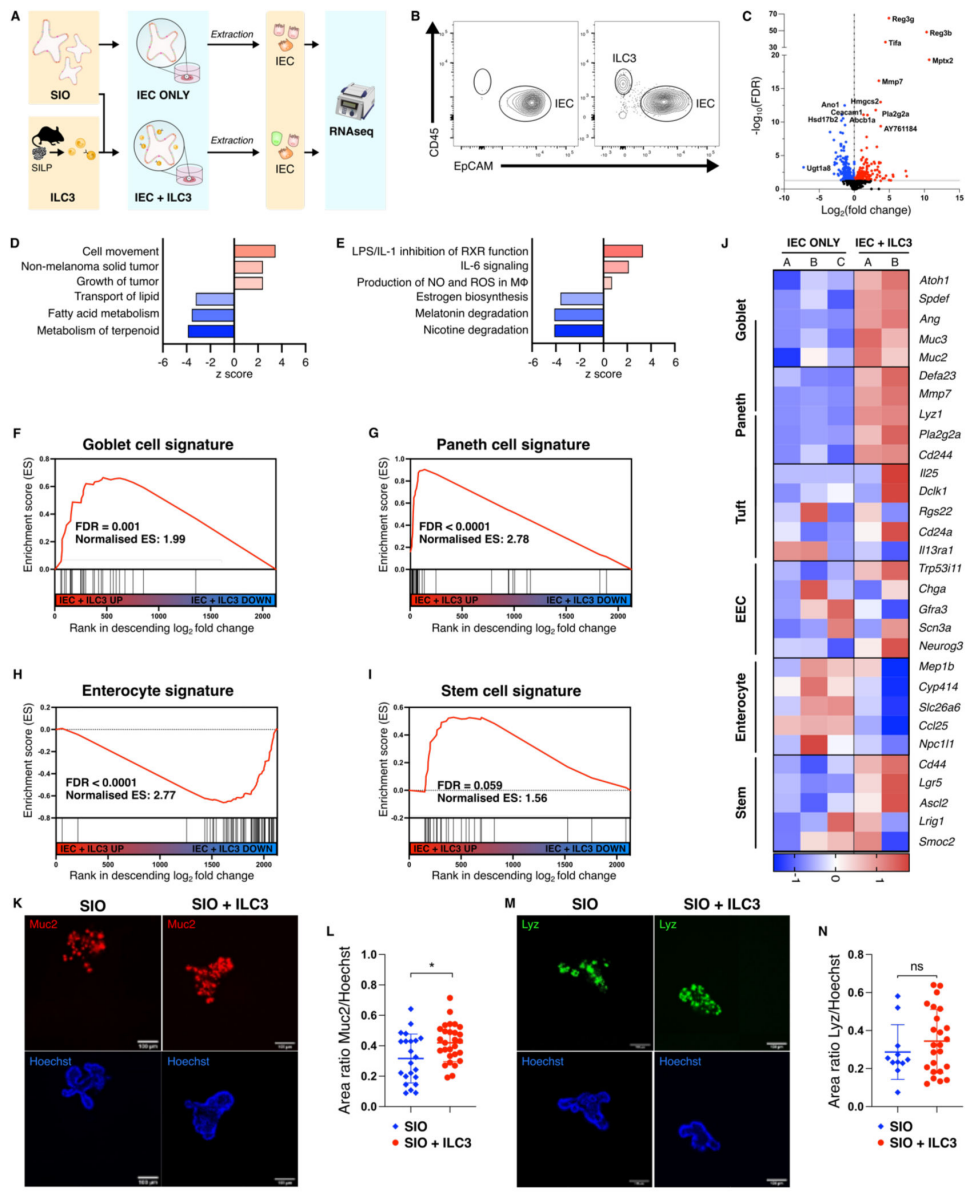


To discern the impact of ILC3 on epithelial phenotype, we optimised co-cultures of SIO with ILC3 to sustain viability of both IEC and ILC3 for 4 days, sufficient time to capture sustained impact of ILC3 on a full cycle of epithelial differentiation from the Lgr5^+^ stem cell crypt. SIO co-cultures were established with a heterogenous population of ILC3 (SFig. 1A), which include CCR6^-^ NKp46^+/-^ subsets and CCR6^+^ post-natal LTi-like cells, to create a representative model of the ILC3 compartment. Viability of ILC3 in SIO co-culture was comparable to ILC3 suspension culture, however there was a small (~2%) reduction in the viability of ILC3 when cultured alone in Matrigel (SFig. 1B). Next, we compared the transcriptome of IEC isolated from SIO in co-culture with ILC3 (IEC + ILC3) to those cultured without ILC3 in the same culture conditions (IEC ONLY), as previously described for ILC1 co-cultures^21^ (Fig. 1A-B, SFig. 1C-D). In this dataset, 495 transcripts were significantly differentially regulated between IEC ONLY and IEC + ILC3 (Fig. 1C, FDR < 0.1; red for upregulated genes, blue for downregulated). Ingenuity Pathway Analysis (IPA) predicted upregulation of cell movement and tumour associated functions, as well as downregulation of metabolic pathways and lipid transport in IEC + ILC3 (Fig. 1D-E). Gene Set Enrichment Analysis (GSEA) of cell-type specific signatures^22^ revealed significant enrichment of secretory goblet and Paneth cell signatures in IEC + ILC3 (Fig. 1F-G), which was accompanied by a decrease in enterocyte profiles (Fig. 1H). Stem cell signatures were unaltered in IEC + ILC3 (Fig. 1I). Expression of several canonical goblet (e.g *Spedf, Muc3, Muc2*) and Paneth (*Defa23, Mmp7, Lyz*) cell markers were increased in IEC + ILC3 when compared to IEC ONLY (Fig. 1J). Muc2 enrichment was also detected by immunofluorescence when using mCherry-MUC2 expressing transgenic mouse organoids that were co-cultured with ILC3s when compared to mCherry-MUC2 organoids cultured alone (Fig. 1K, L). However, despite observing a higher tendency within Lysozyme staining area for Paneth cells, we did not observe any significant increase (Fig. 1M, N). We therefore conclude that ILC3 promote enrichment of some secretory intestinal epithelial signatures in an SIO model system.

### Goblet cells maintain NKp46^+^ ILC3 in co-culture and promote IL-22 production

**Figure 2:**
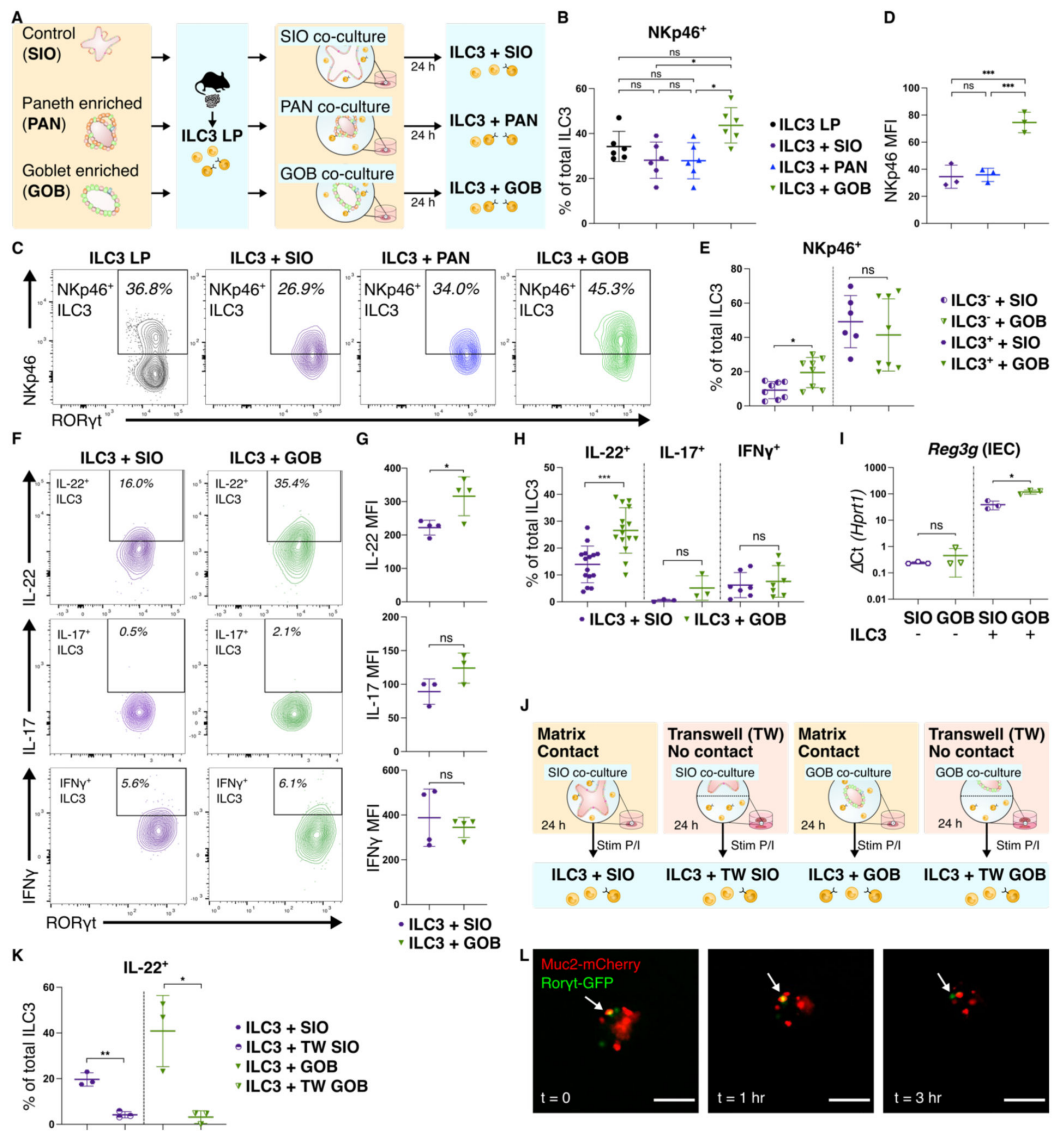


Given the upregulation of secretory IEC signatures by ILC3, we next sought to characterise how these secretory cells may impact ILC3 in co-culture (Fig. 2A). In conventional SIO culture, the proportion of Paneth and goblet cells is reduced when compared to primary tissue (SFig. 2A). We therefore generated SIO enriched for Paneth cells (Paneth cell enriched SIO, PAN) and goblet cells (goblet cell enriched SIO, GOB), modified from established protocols^23^ (SFig. 2B). Differential expression of *Lyz1* and *Muc2* was seen in PAN and GOB respectively, validating these enrichment processes (SFig. 2C). NCR^+^ ILC3 subsets are of interest as important homeostatic producers of IL-22^24^ and for the reduced proportion of these cells in the intestinal mucosa of individuals with IBD^16–18^. Following co-culture with GOB we observed a significant increase in the proportion of NKp46^+^ ILC3 when compared to SIO or PAN co-culture, with the proportion of NKp46^+^ ILC3 comparable to that seen in LP ILC3 (Fig. 2B-C, SFig. 2D). Expression of NKp46 in ILC3 was higher following GOB co-culture when compared to SIO (Fig. 2D), as expected by the increased proportion of NKp46^+^ ILC3. We then separately seeded NKp46^-^ and NKP46^+^ ILC3 into co-culture with SIO or GOB (SFig. 2E). When compared to conventional co-culture with SIO, GOB only increased the proportion of NKp46^+^ ILC3 in co-cultures seeded with NKp46^-^ ILC3, but not NKp46^+^ ILC3 (Fig. 2E). This was not driven by a change in the total number of ILC3 obtained from co-cultures (SFig. 2F). This suggests that the GOB promote expression of NKp46 in NKp46^low^ or NKp46^-^ ILC3.

Given the robust ability of GOB to maintain the proportion of NKp46^+^ ILC3 in co-culture, we next aimed to characterise if goblet cells influence ILC3 cytokine profile (SFig. 2G). In conjunction with IL-22, ILC3 can also produce IL-17 and IFNγ in a subset and stimulation dependent fashion^25,26^. Under non-polarising stimulation conditions, GOB increased the proportion of IL-22^+^, but not IL-17^+^ or IFNγ^+^ ILC3 when compared to co-culture with SIO (Fig. 2F-H). Unlike IL-17 and IFNγ, IL-22 expression increased in ILC3 co-cultured with GOB when compared to SIO (Fig. 2G). IL-22 has a number of known epithelial transcriptional targets, including the Reg3 family of AMPs^27^, which reinforce the mucosal barrier. To assess the possible feedback of goblet cell induced IL-22 on the epithelium, EpCAM^+^ IEC and CD45^+^ RORγt^+^ ILC3 were FACS purified from SIO and GOB cultures and ILC3 co-cultures respectively (SFig. 2H). As expected from our previous data, *Il22* transcripts were increased in ILC3 co-cultured with GOB when compared to SIO (SFig. 2I). Concomitantly, expression of the IL-22 target gene *Reg3g* was higher in both SIO and GOB IEC isolated from ILC3 co-cultures in comparison to culture without ILC3 (Fig. 2I), which is consistent with our RNAseq dataset (Fig. 1C). Notably, *Reg3g* expression was highest in IEC from stimulated GOB-ILC3 co-cultures (Fig. 2I). These results suggest that goblet cells maintain NKp46^+^ expression and promote IL-22 production, which can feedback to upregulate epithelial protective barrier components. To determine if goblet cell regulation of ILC3 phenotype was mediated by soluble factors, co-cultures were established in a porous transwell system that physically separates organoids and ILC3 (Fig. 2J). IL-22 expression by ILC3 was reduced in SIO and GOB transwell co-cultures when compared to matrix co-cultures (Fig. 2K, SFig. 2J), suggesting the importance of physical contact in epithelial mediated regulation of ILC3 derived IL-22. Through live imaging of co-cultures established with RORγt^GFP^ ILC3 and SIO derived from mCherry-MUC2 mice, co-localization of ILC3 and Muc2^+^ cells was detected in our system (Fig. 2L, supplementary video 1). We sought to identify this interaction in mouse small intestinal tissue and we observed wheat germ agglutinin (WGA)^+^ goblet cells in close proximity to CD3^-^ RORγt^+^ ILC3 suggesting that ILC3 may physically interact with goblet cells *in vivo* (SFig. 2K).

### ILC3 and intestinal epithelial cells interact via the Notch pathway

**Figure 3:**
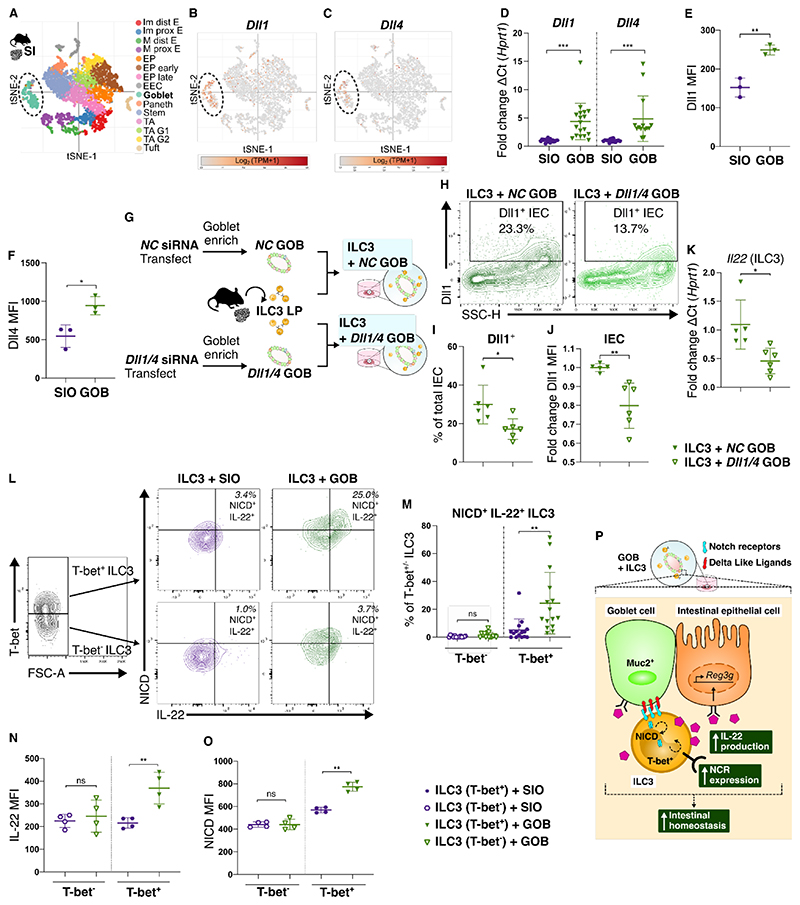


As Notch signalling is dependent on physical interaction^28^, we hypothesised that epithelial modulation of ILC3 in our system was driven by the Notch pathway. Metanalysis of available scRNAseq of the murine small intestinal epithelium^22^ confirmed that goblet cells are enriched for the Notch ligands Delta-Like-Canonical-Notch-Ligand (Dll) 1 and Dll4 (Fig. 3A-C). Expression of *Dll1* and *Dll4* is increased in GOB when compared to conventional SIO (Fig. 3D-F). Metanalysis of a publicly available bulk RNAseq dataset of PAN and GOB^29^ revealed that *Dll1* is enriched in GOB, but not PAN, when compared to conventional SIO (SFig. 3A), offering a potential mechanism for the difference in IL-22 upregulation in PAN and GOB co-cultures (Fig. 2B-D). To validate the importance of goblet cell derived Notch ligands in regulation of ILC3 derived IL-22, we employed siRNA mediated knockdown of Dll in GOB prior to co-culture (Fig. 3G, SFig. 3B). In co-cultures, the proportion of Dll1^+^ IEC and fold change in Dll1 protein expression was reduced in GOB following siRNA mediated knockdown (Fig. 3H-J). Importantly, ILC3 isolated from these cultures had significantly reduced *Il22* expression when compared to those in co-culture with GOB transfected with non-targeting Negative Control (NC) siRNA (Fig. 3K). It is important to note that the siRNA knockdown could have an impact on the number and composition of cells in the organoids. Nevertheless, these data suggest that the epithelium, particularly goblet cells, act as a source of Notch ligands in the intestine to regulate ILC3.

The transcription factor T-bet is required for the development and maintenance of NKp46^+^ ILC3 subsets^30^. Although not yet fully defined, there is an intersection between Notch and T-bet in regulating NCR^+^ ILC3^31–33^. We found Notch1 extracellular receptor expression is enriched in T-bet^+^ NKp46^+^ ILC3 in the SILP (SFig. 3C). To determine the role of T-bet in Notch1 mediated upregulation of IL-22 by GOB in our system, we quantified active Notch1 signalling via intracellular flow cytometric analysis of the cleaved Notch1 Intracellular Domain (NICD), which is generated following Notch1 receptor activation (SFig. 3D). We observed an increase in NICD^+^ IL-22^+^ double positive cells in T-bet^+^ ILC3, but not T-bet^-^ ILC3, in co-culture with GOB (Fig. 3L-M). Protein expression of IL-22 and the abundance of cleaved NICD was increased specifically in T-bet^+^ ILC3 by GOB co-culture (Fig. 3N-O). These results highlight the key subset-specific role for the Notch pathway in regulating ILC3 function, whereby T-bet^+^ ILC3 subsets are sensitive to goblet cell derived Notch signals and respond via production of IL-22 (Fig. 3P).

**Figure 4:**
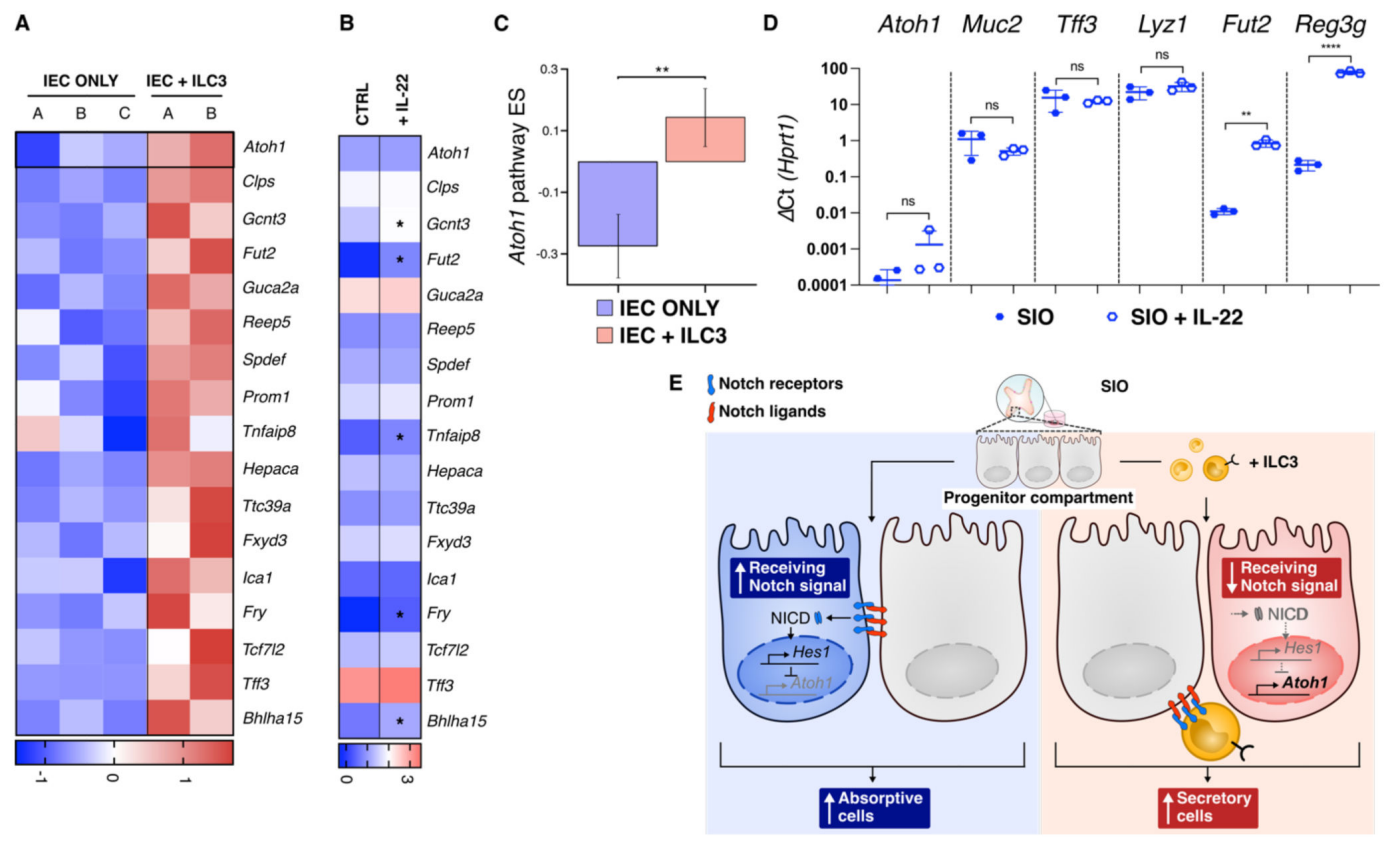


We next considered the possible contribution of Notch mediated interaction between ILC3 and the epithelium to the skewed differentiation of secretory cells initially observed in our transcriptional dataset (Fig. 1). Notch signalling is important in secretory epithelial lineage determination, through Dll and Notch receptor mediated lateral inhibition of the secretory transcription factor Atoh1^34^. Gene set variation analysis (GSVA) of a defined murine Atoh1 targetome^35^ revealed significant enrichment of *Atoh1* and *Atoh1* target genes in IEC + ILC3 (Fig. 4A-C), a signature not described to the same extent in literature supplementing IL-22 into organoid cultures in the absence of ILC3. We did not see upregulation by RT-qPCR of *Atoh1*, the goblet cell markers *Muc2* or *Tff3* or the Paneth marker *Lyz1* following 24 hours of SIO treatment with 10 ng/ml IL-22, although upregulation of *Fut2* and *Reg3g* was seen (Fig. 4D). This is in line with recent findings which use cytokine addition to SIO to describe dichotomous goblet cell responses to IL-13 and IL-22^9^. Thus, we hypothesise a mechanism by which ILC3 may skew secretory vs absorptive differentiation through binding of Notch ligands in progenitor epithelial populations (Fig. 4E).

Utilising a reductionist co-culture system, we describe two new complimentary Notch mediated signalling modules between the epithelium and ILC3. Although the Notch pathway is known to play a role in the maintenance of NCR^+^ ILC3, this has largely been determined through the use of feeder cell lines engineered to express Dll1 and Dll4 (OP9-DL1, OP9-DL4), which are not representative of intestinal tissue^31–33^. As such, it has remained unclear what cellular sources provide these ligands to ILC3 within the primary intestine, and teasing this apart in the multifactorial and multicellular *in vivo* environment is challenging. The reductionist organoid model we describe has provided an elegant approach to identify the epithelium, particularly goblet cells, as a potential local intestinal source of Notch ligands for ILC3. A caveat to our findings is that GOB may not be exclusively enriched for goblet cells (as previously described^29^), and the possible contribution of other epithelial cell types cannot be entirely excluded. Notch ligands are also expressed by other non-epithelial cells in the small intestinal microenvironment, including stromal fibroblasts^36^, with further work needed to define the contribution of other cellular sources of Notch ligands in regulating ILC3. Our results do however add detail to the description of Notch in subset specific regulation of ILC3, highlighting the ability of the epithelium to promote IL-22 specifically in T-bet^+^ ILC3 via Notch1 activation. This is likely regulated by complex epigenetic and chromatin remodelling machinery, with an interesting candidate for future research being the catalytic subunit of the chromatin-remodelling BAF complex Brahma-related gene 1 (Brg1), which promotes expression of *Tbx21* in ILC3 through histone modification and is necessary for OP9-DL4 mediated upregulation of NKp46 in cultured ILC3^31^.

The enrichment of DLL1 in human intestinal goblet cells (SFig. 4) provides exciting potential translation of the mechanism we describe. This could be particularly pertinent in the context of IBD, where a reduction in *DLL1* expression within goblet cells has been reported^37^. Our work postulates a causative mechanistic link between a decrease in goblet cells and the characteristic loss of NCR^+^ ILC3 observed in this disease^16–18^. Furthermore, the ability of ILC3 to directly skew absorptive versus secretory epithelial lineage dynamics could also have substantial implications in IBD, as absorptive intestinal epithelial progenitors are significantly enriched in some IBD cohorts, which may degrade barrier function through loss of protective secretory populations^13^. Overall, our findings further demonstrate organoids as a powerful tool to study epithelial-immune interactions^38^. The exquisite amount of experimental control offered by this system can continue to develop our understanding epithelial-ILC interactions in fine detail, to outline mechanisms that may underpin intestinal homeostasis and be dysregulated in clinically relevant diseases.

## Methods

### Animals

CD45.1, C57BL/6J B6.SJL^PtprcaPepcb/BoyCrl^ (CD45.1) mice were a generous gift from Professor T. Lawrence. RORγt–GFP (green fluorescent protein, RORγt^GFP^), C57Bl/6J reporter mice were a generous gift from G. Eberl^39^. All animals were maintained at Charles River and in the New Hunt’s House King’s College London animal facilities by Biological Services Unit staff. Animals were maintained with enrichment in specific pathogen-free conditions with a 12-h light/12-h dark cycle, at ~19–22 °C and ~50% humidity in accordance with the UK Animals (Scientific Procedures) Act 1986 (UK Home Office Project License PPL:70/7869 to September 2018; P9720273E from September 2018).

### SIO establishment and culture

SIO cultures were established from the distal (ileum) small intestine of female 6-10 week old CD45.1 mice and maintained in Matrigel (Corning) or Cultrex (R&D) following established protocols^40^. SIO were propagated in basal medium (Advanced DMEM/F12 (Adv-F12), 2 mM GlutaMAX, 10 mM HEPES, 1× antibiotic– antimycotic, 1× N-2 supplement, 1× B-27 supplement, all Thermo Fisher; 1mM acetyl-l-cysteine, Sigma) supplemented with 50 μl/ml from both R-spondin (RSpo1-Fc) and Noggin cell lines and epidermal growth factor (50 ng/ml, R&D), termed ENR media. SIO were passaged every 4-6 days by mechanical disruption and cultured at 37 °C, 5% CO_2_ as previously described^40^. The RSpo1-Fc cell line was a gift from C. Kuo (Stanford University, USA) and the Noggin cell line was a gift from the Hubrecht Institute.

Paneth cell enriched (PAN) and goblet cell enriched (GOB) SIO were generated following a protocol modified from^23^. Briefly, organoids were mechanically disrupted and seeded into media containing 3 μM CHIR99021 (Axon) and 1 mM Valporic acid (Sigma) to expand the stem cell compartment. After 24 hours organoids were washed (without disrupting the matrix bubble) 2-3 times in Adv-F12 for 3-4 minutes, to thoroughly remove stem cell media. Media was changed to either ENR media (for conventional SIO), or additionally supplemented with 3 μM CHIR99021 and 10 μM DAPT (Sigma) for Paneth cell enrichment (PAN) or 2 μM IWP-2 (Sigma) and 10 μM DAPT for goblet cell enrichment (GOB).

For IL-22 treatment, organoids were harvested and passaged as above. Following 24 hours media was removed and replaced with ENR media supplemented with 10 ng/ml recombinant murine (rm)IL-22 (R&D). Organoids were harvested after 24 hours for analysis by RT-qPCR.

### Isolation of ILC3 from the murine lamina propria

ILC3 were isolated from the SILP of littermate 6-12 week old female RORγt^GFP^ mice following established protocols^41^. Briefly, intestines were harvested, and mesenteric fat and Peyer’s Patches removed. Tissue was then opened longitudinally and thoroughly cleaned in ice-cold PBS to remove luminal material. 0.5 cm-1 cm intestinal sections were incubated in epithelial cell removal buffer (5 mM EDTA and 10 mM HEPES in Hanks’ balanced salt solution (HBSS), Gibco) for 2 × 15 min with gentle shaking at 37 °C. Following a series of washes in ice-cold PBS, tissue was minced for extensive digestion in digestion buffer (collagenase (500 μg/ml), dispase (0.5 U/ml), DNase1 (500 μg/ml), 2% fetal bovine serum (FBS) in HBSS) for 2 × 15 min with gentle shaking at 37 °C. Digested samples were then filtered through a 40 μm strainer in neutralisation buffer (DMEM (Sigma), 20% FBS, 20 μM β-mercaptoethanol (R&D)) and lymphocytes isolated through 80%-40% isotonic Percoll density gradient separation via centrifugation for 25 min at 900 x *g* with no acceleration or deceleration breaks. The lymphocyte laden interphase between 40%-80% Percoll was collected in ice-cold PBS and filtered through a 40 μm strainer. Lymphocytes were then rinsed with PBS and stained with fixable LIVE/DEAD Fixable Blue Dead Cell Stain (ThermoFisher) for 15 min in the dark at 4 °C according to manufacturer´s instructions. Dye was quenched with PBS2 (PBS (Gibco), 2% FBS) and Fc receptor blockade following incubation with 0.25 mg/ml anti-CD16/CD32, clone 93 (2B Scientific) for 10 min at 4 °C. Extracellular staining was then completed following standard flow cytometry protocols (1 μl antibody/100 μl sorting buffer/5 million cells), incubation with antibody for 30 min in the dark at 4 °C, prior to rinsing and resuspension in PBS2. Antibodies for ILC3 isolation were as follows: CD3–Fluor450 (RB6-8C5), CD5–Fluor450 (53-7.3), CD19–Fluor450 (eBio1D3), Ly6G–Fluor450 (RB6-8C5), CD45–BV510 (30-F11, BioLegend) CD127–APC (A7R34), NKp46–PE/Cyanine7 (29A1.4), all eBioscience (with the exception of CD45). ILC3 were defined as CD45^+^, Lineage^-^ (CD3e, CD19, CD5, Ly-6G/Ly-6C), CD127^+^, RORγt^+^ and additionally NKp46+/- where defined. ILC3 were sorted on a 70 μm nozzle using a BD FACSAria II/III into ice-cold Adv-F12 and used immediately for co-culture experiments.

### Establishing ILC3-organoid co-cultures

Co-cultures between ILC3 and organoids were established following a modified protocol from^42^. In short, 5000-10,000 sorted ILC3 were centrifuged (300 x *g* for 5 min at 4 °C) with freshly disrupted SIO (for RNAseq experiments), or 100-150 3 day old SIO, PAN or GOB, resuspended in 15-30 μl of Matrigel or Cultrex and plated on a 48-well or round bottom- 96 well plate. Cultures were incubated at 37 °C, 5% CO_2_ in ENR media supplemented with 50 mM β-mercaptoethanol, 20 ng/ml rhIL-2 (Sigma) and 20 ng/ml rmIL-7 (R&D). Co-cultures for RNAseq experiments were additionally supplemented with 1 ng/ml IL-15 (R&D), and harvested for analysis following 4 days of co-culture. Otherwise, co-cultures were analysed following overnight culture.

### Cell isolation from co-cultures

Co-cultures were processed for FACS purification or flow cytometric analysis following protocols modified from^21^. For stimulation, following 24 hours co-cultures were stimulated with 10 ng/ml phorbol myristate acetate (Sigma) and 1 μM ionomycin (Sigma) (P/I) for 4-5 hours. 2 μg/ml Brefeldin A (eBioscience) and 2 μM Monensin (Sigma) was added at the time of stimulation. To harvest co-cultures for FACS purification or flow cytometric analysis, matrix bubbles were rinsed in PBS and dissociated either in 15 ml tubes or the plate (if cultures established in round bottom 96-well plate) via addition of TrypLE (Gibco) + DNAse (250 μg ml) for 20 min at 37 °C. Cultures were then mechanically disrupted to obtain single cells and rinsed in PBS2 prior to filtration through a 70 μm strainer. Cells were then processed for live/dead staining, Fc receptor blockade and extracellular staining as described above. Extracellular antibodies used were as stated above, without addition of CD127-APC and with addition of EpCAM-APCcy7 (G8.8), Dll1-APC or -PE (HMD1-3) and Dll4-APC or PE (HMD4-1) (all BioLegend) as stated.

For FACS purification of IEC and ILC3 for downstream analysis by RT-qPCR, cells were sorted (as described above) into Buffer RLT (Qiagen) plus β-ME and immediately frozen on dry ice and stored at -80 °C.

For flow cytometric analysis, cells were either run immediately on a BD Fortessa 2/3 (if only extracellular staining was performed) or processed for intracellular staining. Here, cells were rinsed in PBS and processed for intracellular staining using the Foxp3 staining kit (Thermo Fisher Scientific) following manufacturer´s protocols. In short, cells were fixed and permeabilised prior to intracellular staining for 1-2 hours at room temperature in the dark.

Cells were then rinsed and analysed on a BD Fortessa 2/3. Antibodies used as stated for intracellular staining were as follows: IL-22-APC (IL22JOP, eBioscience), IL-17A-PE/Dazzle (TC11-18H10.1, BioLegend), IFN-γ- PerCPeFluor710 (XMG1.2, eBioscience), RORγt-BV786 (B2D, Invitrogen), T-bet-BV711 (4B10, BioLegend), NICD-PE (mN1A, BioLegend).

### RNAseq dataset production and analysis

The RNAseq dataset presented was generated concurrently with our previously published dataset describing changes to IEC transcription in SIO co-cultured with ILC1^21^. As previously described^21^, SIO were harvested isolated by FACS as described above into RLT (QIAGEN) lysis buffer. RNA was harvested using an RNeasy MicroRNA isolation kit (QIAGEN), and RIN values were assessed using an RNA 6000 Pico Kit (Agilent). The library was prepared using SMART-Seq2 and sequenced using an Illumina HiSeq 4000 at the Wellcome Trust Oxford Genomics Core, where basic alignment (GRCm38.ERCC (2011)) and quality control were also performed. Raw data in FASTQ files were analysed by FastQC v0.11.8 to identify quality issues. Acceptable data were trimmed by Trimmomatic 0.39 to remove poor quality reads and adapter sequences. Processed transcripts were indexed and aligned to GRCm38.p6 v98 from Ensembl using Salmon 0.14.1 to quantify transcript-level read counts, which were summarised to gene-level counts by tximport 1.13.16. Differential gene expression analysis (DGEA) was performed using DESeq2 1.25.13 on R 3.6.1. Results were imported into Ingenuity Pathway Analysis (Qiagen) Fall 2019 release for further functional analyses. Gene set enrichment analysis (GSEA) was performed using customised gene sets for specific cell types on the software (Broad Institute)^43,44^. These customised gene sets were comprised of the top 100 differentially expressed genes for enterocyte, goblet cell, Paneth cell, stem cell, tuft cell and enteroendocrine cells from the single cell transcriptome of mouse small intestinal epithelium^22^. Gene set variation analyses (GSVA)^45^ was performed using a Atoh1 targetome gene set defined by unbiased genome wide approaches of the murine transcriptome^35^.

### RT-qPCR

Cells were harvested and rinsed in PBS prior to lysis in Buffer RLT (Qiagen) plus 10 μl/ml β-ME. RNA was extracted using an RNeasy MiniRNA isolation kit (QIAGEN) and complementary DNA libraries produced using RevertAid (Fisher) with random hexamer primers. RT-qPCR was completed using Fast SYBR Green Mix (Applied Biosystems) run on a CFX384 Touch real-time PCR detection system (Bio-Rad) or TaqMan Gene Expression Master Mix (Applied Biosystems) with fluorescein amidite probes. All kits were used following manufactures instructions. Samples were run in technical triplicate, with no template controls and melting curves included for quality control. ΔCt values were normalized to the housekeeping gene *Hprt1*. Primers were verified via BLAST against the *Mus musculus* genome via ensemble.org. Primer sequences are outlined in Supplementary Table 1.

### siRNA transfection

SIO were harvested and mechanically disrupted as for passage. Transfection was completed with the TriFECTa RNAi Kit (IDT) and Lipofectamine 2000 (Invitrogen), following manufacturer instructions, and using a protocol modified from^46^. Dicer-substrate siRNAs (DsiRNAs) were acquired from IDT (Dll1: mm.Ri.Dll1.13.1, mm.Ri.Dll1.13.2 and mm.Ri.Dll1.13.2. Dll4: mm.Ri.Dll4.13.1, mm.Ri.Dll4.13.2 and mm.Ri.Dll4.13.3. Non-targeting: Negative Control DS NC1) using a predesigned and computationally validated library. 100-200 crypt structures were transfected with the appropriate siRNA complexes to a final concentration of 1 μM in transfection media (Adv-F12, 10 mM HEPES, 1 mM sodium pyruvate), via centrifugation at 300 x *g* for 5 min at 37 °C, prior to 4 hour incubation at 37 °C, 5% CO_2_. Crypt structures were then rinsed in Adv-F12, plated in Matrigel, goblet enrichment and ILC3 co-culture performed as previously described.

### Immunofluorescence of mouse intestinal organoids

For live imaging of goblet cells and RORyt^+^ ILC3 co-cultures, RORyt^+^ GFP ILC3 were isolated from mouse intestinal lamina propria and co-cultured with organoids from mCherry-MUC2 transgenic mice^47^. Images of living cells incubated at 37°C, 5% CO2 for 2-3 hours were acquired every 5 minutes using Incucyte Live-Cell Analysis System´s 10x objective.

In order to quantify goblet cells and Paneth cells in our co-culture system, freshly disrupted mCherry-MUC2 organoids and SIO were co-cultured for 2 days alone or with ILC3s, removed from Matrigel and fixed with 4% Paraformaldehyde (PFA) for 30 minutes at RT. mCherry-MUC2 organoids were washed with PBS and stained with Hoechst 33342 nucleic acid stain (Invitrogen, H3570) for 30 minutes at RT in the dark. For SIO staining of Paneth cells, organoids were blocked with FBS solution and stained with lysozyme (ThermoFisher, PA5-16668) overnight at 4°C. The following day, organoids were washed and stained with Hoechst (Invitrogen, H3570) for 30 minutes at room temperature in the dark. After several washing steps, organoids were embedded back into Matrigel and visualized with THUNDER. Area percentage for Muc2, Lyz and Hoechst was calculated using Image J.

### Immunofluorescence of mouse small intestinal tissue

Small intestine was harvested from RORγt–GFP C57Bl/6J reporter mice, split longitudinally, washed in PBS and fixed in 4% PFA for 24 hours. Then, the tissue was cryopreserved with a 30% sucrose solution for 24 hours and embedded in OCT. Next, 30μm sections were cut at -20 degrees Celsius using a Leica CM1950 cryostat and stored at -80. Tissues were rehydrated in 0.5% BSA in PBS for 10 minutes and blocked with 5% BSA and 0.1% Triton-X 100 in PBS for 1 hour. Tissues were then stained with anti-GFP (Invitrogen, A21311), anti-mouse CD3-biotin (Invitrogen, 13-0033-82) and anti-WGA (Invitrogen, W2464) overnight at 4°C. The following day slides were washed with PBS 3 times for 5 minutes, then stained with SA-AF647 (BioLegend, 405237) at RT for 1-2 hours. Slides were then washed again and stained with DAPI (Merck, D9542-10MG). After washing them, tissues were mounted with Prolong gold mounting medium. Images were acquired on a Leica TCS SP8 X WLL inverted confocal microscope, with HC Plan Apochromat 63x CS2 (NA = 1.40) oil lens. Images of the tissue were captured at 1024x1024 pixels with line averaging of 3 onto HyD or PMT detectors. Images were analysed on FiJi (ImageJ) software and only the maximum intensity projections of these 3D stacks are shown in the results.

### Statistics and graphics

Flow cytometry data was acquired on a Fortessa II (BD Biosciences) using DIVA software and analysed using FlowJo 10.4.1. Statistical analysis were performed in GraphPad Prism version 8.1.2. Graphics were produced in Inkscape.

## Supplementary Material

Supplementary Materials

## Data Availability

RNAseq data will be made accessible in publicly available databases upon publication.
