# Supplementary material for: Bi-directional signalling between the intestinal epithelium and type-3 innate lymphoid cells (ILC3) regulates secretory dynamics and IL-22
Source: Mucosal Immunol. Author manuscript; Available in PMC 2024 Mar 19. (PMC7615753; doi:10.1016/j.mucimm.2023.11.002)
Supplement: Supplementary Materials [file EMS194712-supplement-Supplementary_Materials.pdf]

SUPPLEMENTAL FIGURES

Species	Gene	Forward primer (5’-3’)	Reverse primer (5’-3’)
Mouse (used for SYBR green amplification)	*Hprt1*	TGGATACAGGCCAGACTTTGTT	CAGATTCAACTTGCGCTCATC
	Gapdh	TGTGTCCGTCGTGGATCTGA	TTGCTGTTGAAGTCGCAGGAG
	Muc2	ATGCCCACCTCCTCAAAGAC	GTAGTTTCCGTTGGAACAGTGAA
	Lyz1	GAGACCGAAGCACCGACTATG	CGGTTTTGACATTGTGTTCGC
	Dll1	TGGCGCAGGCATCGA	GGCGGCTGATGAGTCTTTCT
	Dll4	TGTCTCCACGCCGGTATTG	AGGTCGTCTCCCGGTGTGT
	Reg3g	ATGCTTCCCCGTATAACCATCA	ACTTCACCTTGCACCTGAGAA
	Atoh1	AGATCTACATCAACGCTCTGTC	ACTGGCCTCATCAGAGTCACTG
	Fut2	ACCTCCAGCAACGAATAGTGA	GCCGATGGAATTGATCGTGAA
	Tff3	TTGCTGGGTCCTCTGGGATAG	TACACTGCTCCGATGTGACAG
Mouse (used for TAQ polymerase amplification)	**Gene**	**Probe identifier**	**Source**
	Il22	Mm01226722	ThermoFisher
	Gapdh	Mm99999915	

Supplementary table 1.

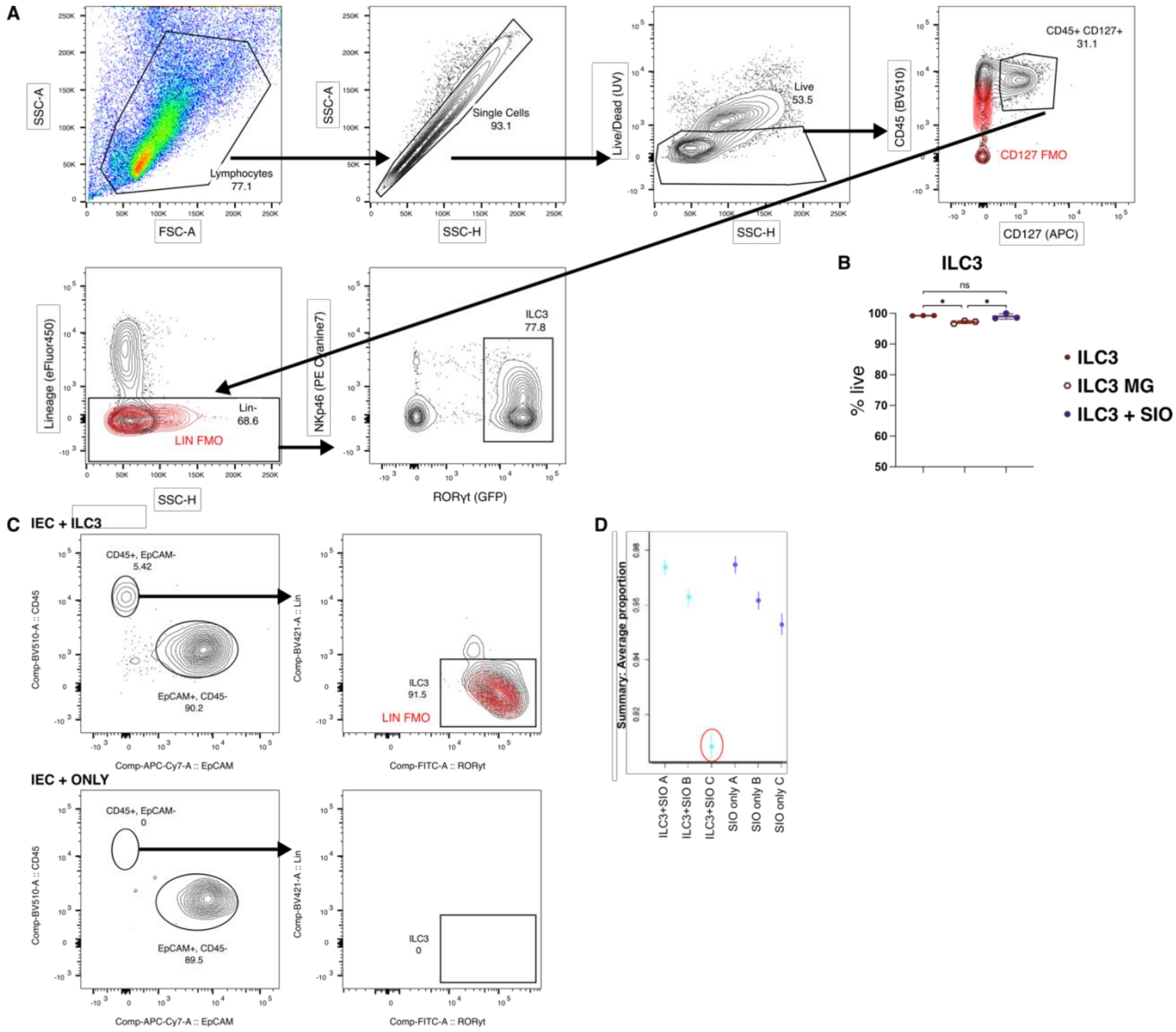

Supplemental figure 1. ILC3 gating strategy, FACS purification of epithelial cells from co-cultures and RNA sequencing quality control.

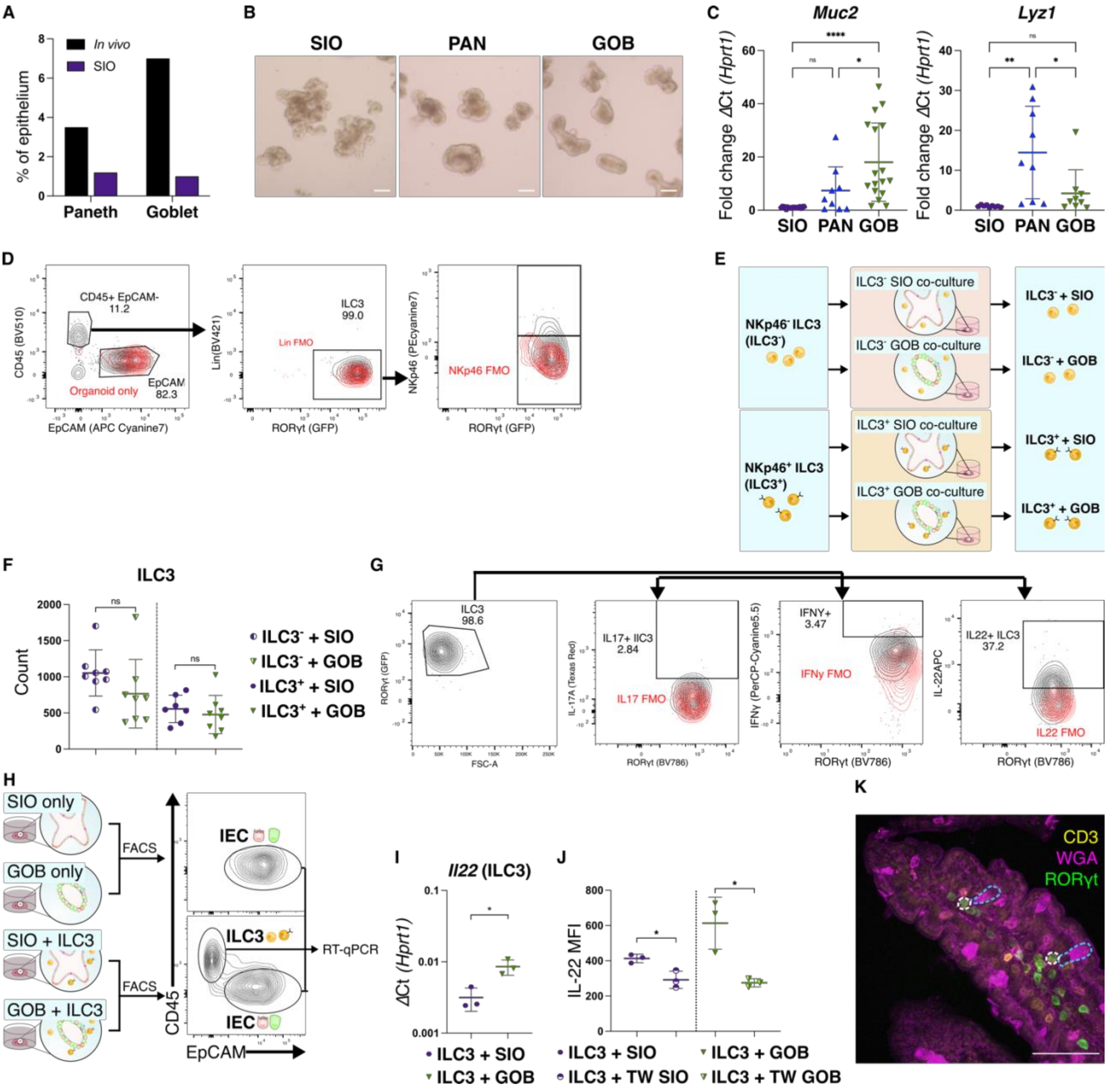

Supplemental figure 2. Goblet and Paneth cell enriched SIO and ILC3 co-culture.

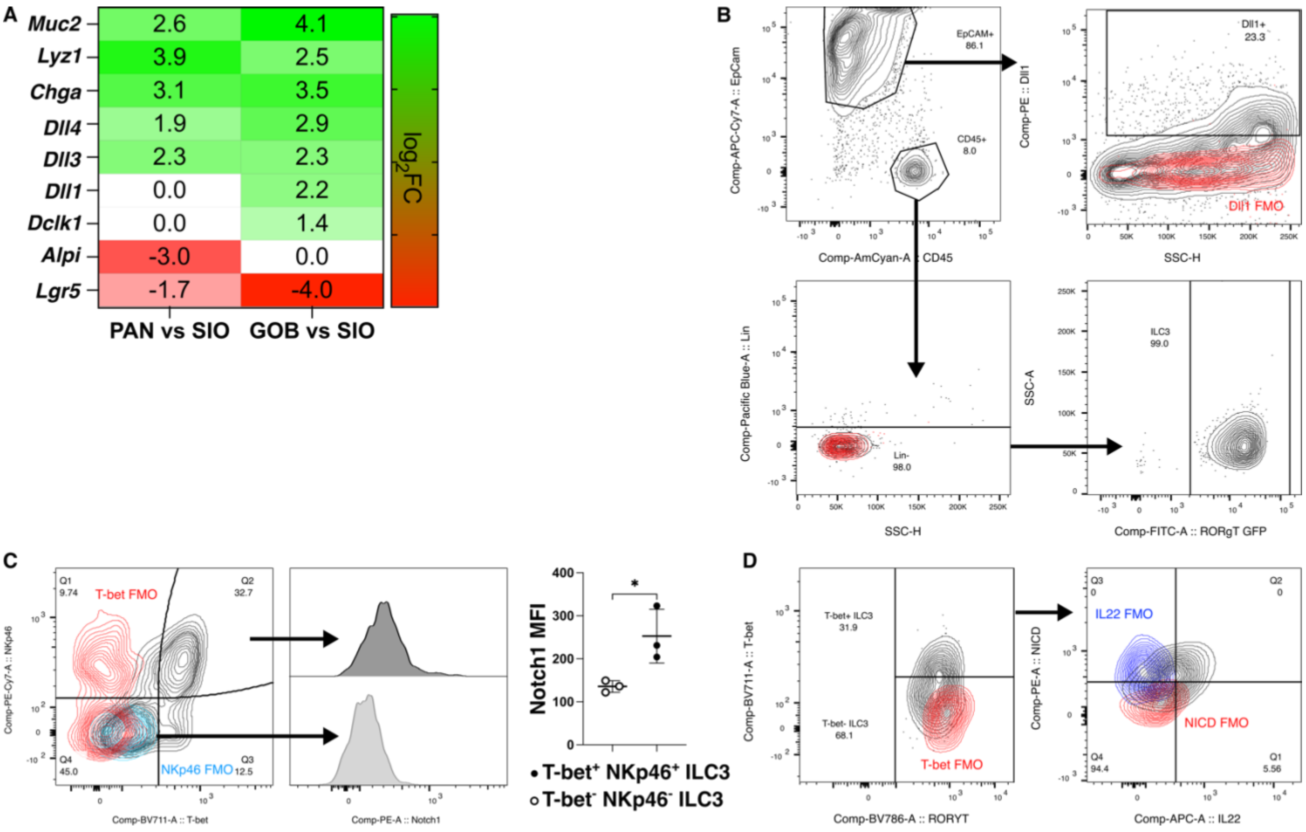

Supplemental figure 3. Investigation of the Notch pathway in GOB-ILC3 co-cultures.

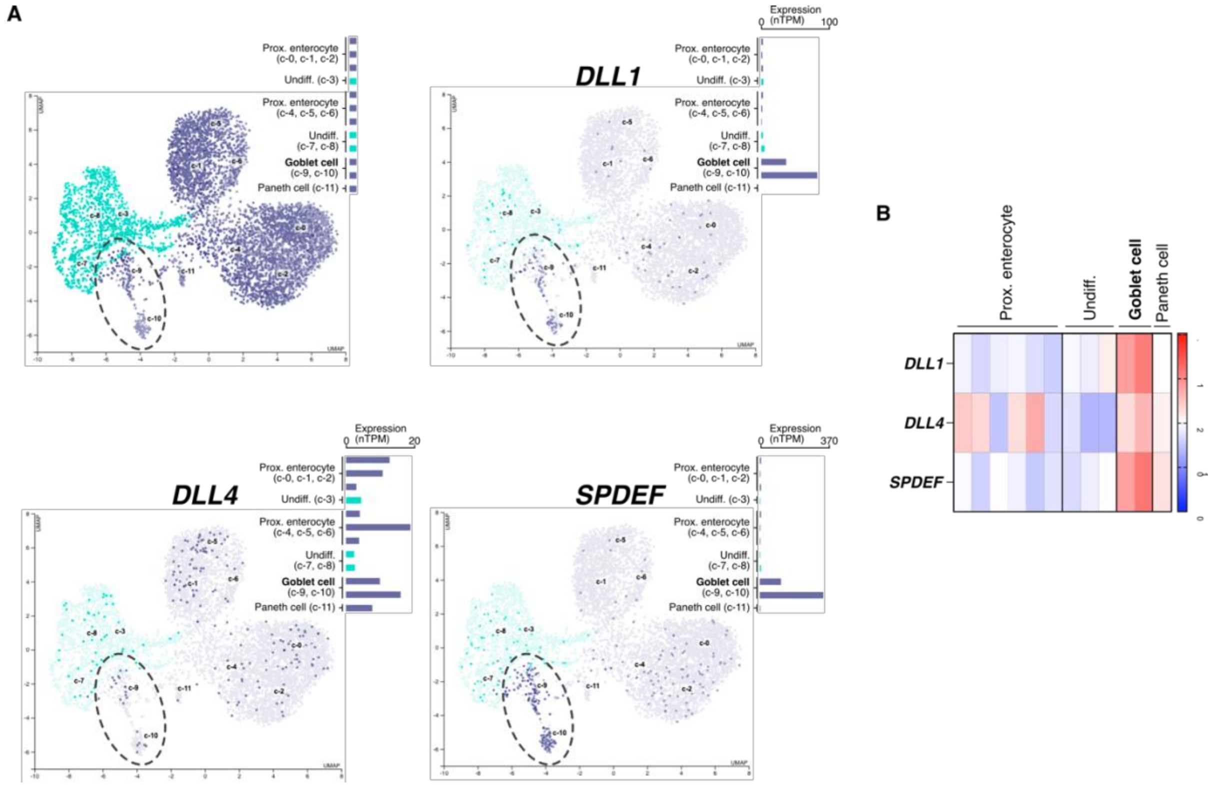

Supplemental figure 4. Enrichment of *DLL1* in human small intestinal goblet cells.
